# Transcriptomic analysis identifies four novel receptors potentially linking endometrial cancer with polycystic ovary syndrome and generates a transcriptomic atlas

**DOI:** 10.18632/oncotarget.28513

**Published:** 2023-09-22

**Authors:** Fatma Alqutami, Mahmood Hachim, Charlie Hodgman, William Atiomo

**Affiliations:** ^1^College of Medicine, Mohammed Bin Rashid University of Medicine and Health Sciences, Dubai Healthcare City, Dubai, UAE; ^2^School of Biosciences, University of Nottingham, Loughborough LE12 5RD, UK

**Keywords:** polycystic ovary syndrome, endometrial cancer, transcriptomics, IGF1, *in-silico*

## Abstract

Polycystic Ovary Syndrome (PCOS) is associated with a 3 to 4-fold increased risk of endometrial cancer (EC), but molecular mechanisms are unclear. Upregulation of the IGF1 gene in PCOS endometrium may increase EC risk, but this is uncertain. We aimed to investigate links between EC and PCOS, by analysing publicly available transcriptomic data. The NCBI Gene Expression Omnibus was used to identify relevant studies. Differentially expressed genes (DEGs) were identified and analysed using Metascape to identify pathways of interest. PCOS DEGs that encode proteins secreted into blood were identified using the Human Protein Atlas blood protein database. EC DEGs that are cellular receptors were identified using EcoTyper. These were intersected to identify which EC receptors interact with PCOS secreted proteins. Seven receptors were identified in EC but only PTPRF, ITGA2, ITGA3 and ITGB4 genes were expressed on epithelial cells. Pathway enrichment of these genes showed that the major and common pathway involved was that of the PI3K-AKT signalling pathway which was consistent with a link between PCOS and EC. However, IGF1 was down regulated in PCOS and EC. These findings hold significant promise for improving our understanding of mechanistic pathways leading to EC in PCOS.

## INTRODUCTION

Endometrial cancer (EC) is the second most diagnosed cancer in women globally, with 417,000 new cases and 97,000 deaths in 2020 [1]. Obesity is a known risk factor for EC and obesity assessed by body mass index (BMI) at diagnosis is associated with worse survival among women diagnosed with EC [2]. EC is histologically divided into two subgroups: endometrioid (type 1) and non-endometrioid (type 2), with the endometrioid group oestrogen-responsive, making up about 80% of cases and often with a good outcome. The non-endometrioid group is less common, less oestrogen sensitive and associated with a poorer outcome [3].

The most frequently altered mutation in type 1 EC, is the PI3K/AKT/mammalian target of the rapamycin (mTOR) (P1K3CA) pathway [4]. Elevated IGF-1 and insulin levels have been shown to directly stimulate cell proliferation through activation of the P1K3CA pathway. Higher levels of phosphorylated IGF-I/insulin receptor gene have also been associated with EC risk factors such as diabetes in postmenopausal women [5]. The precise association between serum IGF-1 and EC is still uncertain and results from case control studies are conflicting [6, 7]. Prospective analyses are more useful than case-control studies when looking at predictors of disease risk, however these are sparse and have shown no association with total IGF1 [8, 9].

Polycystic Ovary Syndrome (PCOS), the most common female endocrinopathy (3.1–20% of women of reproductive age), is associated with obesity, infrequent menstrual periods, infertility, hirsutism, enlarged ovaries with many small follicles on ultrasound imaging, increased risks of type-2 diabetes in later life and a 3 to 4-fold increased risk of EC [10]. Mechanisms thought to increase EC risk in PCOS include raised oestrogen levels, obesity and anovulatory menstrual cycles/lack of progesterone/infrequent shedding of the endometrium but questions have been raised about these hypotheses [11]. Increased EC risk and endometrial dysfunction causing miscarriages in PCOS have also been linked to insulin resistance and the associated elevation in serum IGF1 in PCOS [12]. IGF-1 and IGF-2 have also been shown to promote EC cell proliferation, while metformin inhibited this proliferation. Metformin is a biguanide drug that is a first-line treatment for type 2 diabetes that has beneficial effects on various markers of the metabolic syndrome [13].

IGF1 may therefore increase EC risk in PCOS. However, this requires validation, and it is important to investigate potential confounders which affect insulin resistance (e.g., obesity). In a pilot-study [14] in Nottingham, UK, in which 102 women were recruited, they found for the first time that IGF1, IGFBP1 and PTEN gene expression were significantly up-regulated in the endometrium of PCOS, and EC women compared to controls [14]. The changes in gene expression were independent of BMI, waist hip ratio, oestradiol, and androgen levels. Protein validation test in the serum samples in the three groups were consistent with the gene findings. Genes related to the IGF1/IGFBP pathway may therefore contribute to increased risk of EC in women with PCOS. There is however a need for additional validation of these findings given the previous reports showing that serum IGF-I, IGFBP-1, IGFBP-3, and insulin levels seem unrelated to EC risk [7].

There is thankfully now an increasing amount of publicly available transcriptomic data from studies carried out in women with PCOS and endometrial cancer. Bioinformatic analyses of these data offer the potential to investigate research questions, without the need for *in-vivo* studies. The original aim of this study was to investigate the links between EC and PCOS, by analysing publicly available transcriptomic data and investigate IGF-1 and IGFBP gene expression in the endometrium of women with PCOS and EC compared with normal endometrium. Our conclusions at this stage do not support a link between IGF-1 and IGFBP genes in PCOS and EC. However, we have identified four novel receptors which may underpin the risk of EC in PCOS, and we believe our findings provide sufficient evidence to form the basis for a transcriptomic atlas to underpin future research into the links between PCOS and EC and the molecular mechanisms underpinning both diseases.

## RESULTS

At the time of search, NCBI GEO search yielded 813 results for the keyword ‘polycystic ovary’ and 3021 results for ‘endometrial cancer’ in Homo sapiens datasets. Of the polycystic ovary results, 37 were expression profiling by array and 18 were by high throughout sequencing, while in endometrial cancer there were 90 expression profiling by array and 58 by high throughput sequencing. Furthermore, of the 127-expression profiling by array studies identified, only 11 were of patients and healthy controls without or prior to any drug treatment.

### Several differentially expressed genes in polycystic ovary syndrome patients encode proteins secreted into the blood which have receptors in endometrial cancer cells

The cell sources and location of DEGs in polycystic ovary syndrome and endometrial cancer are different. For PCOS DEGs to exert their effect on endometrial cancer, these cells must secrete proteins into the blood that leaves the ovary and goes to the epithelial lining of the uterus. The identified DEGs of PCOS were checked against the protein atlas blood secretome database and protein coding genes were identified (Supplementary Table 1) as well as the corresponding epithelial receptors for the DEGs from endometrial cancer (Supplementary Table 2).

**Figure 1:**
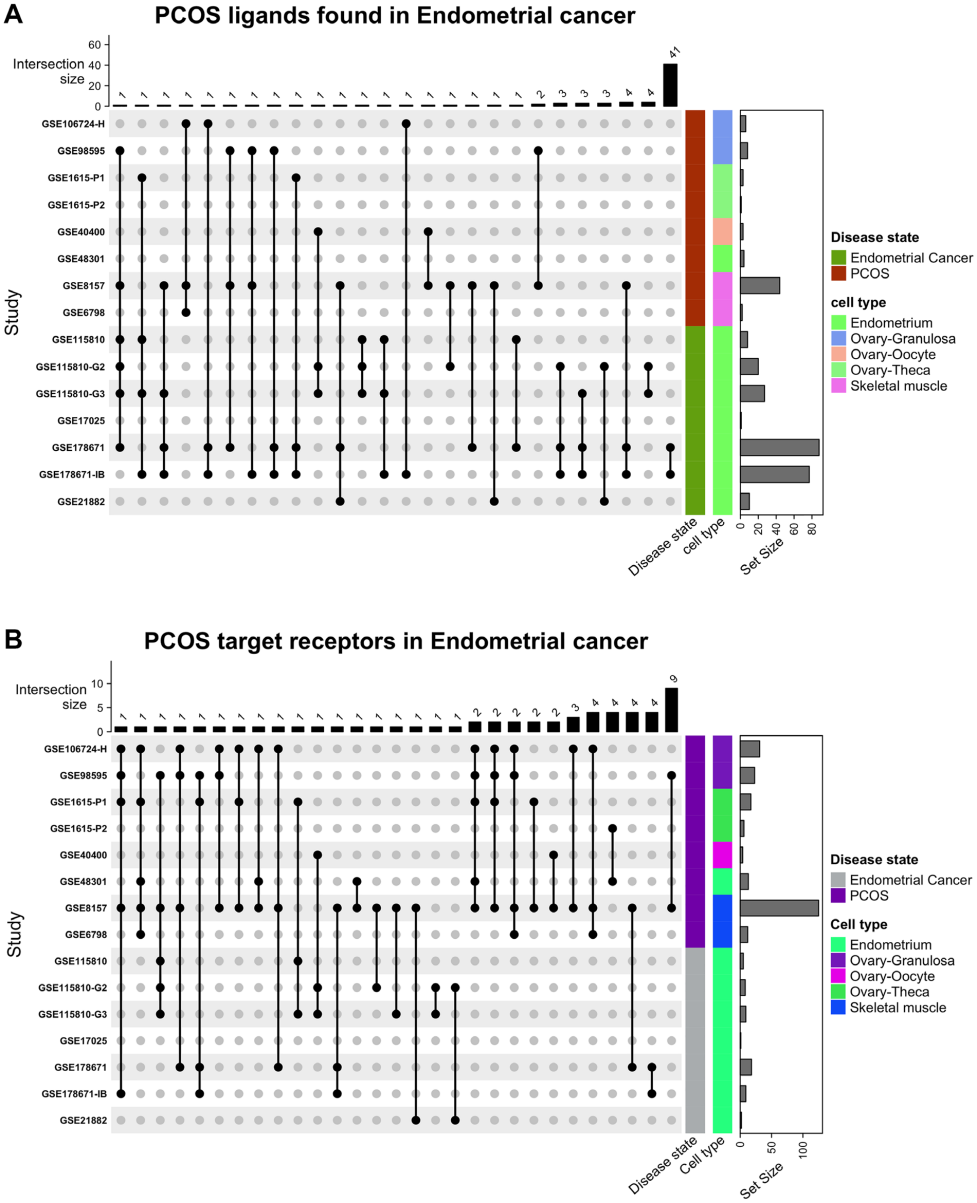
Intersecting the data for all (**A**) ligands from PCOS with its receptors in EC and (**B**) receptors found in endometrial cancer with ligands in PCOS.

The protein encoding DEGs identified in PCOS were intersected with EC receptor DEGs to identify interacting ligands (Figure 1A). There were over 20 different combinations, with most intersections including one gene and several spanning more than one gene (Supplementary File 1 and Table 4 within Supplementary File 1). Most of the intersections occurred within the same disease state rather than between PCOS and EC. Similarly, the receptors found to be differentially expressed in EC were linked to that of the receptors targeted by PCOS ligands (Figure 1B). There were 14 different intersections, with many ligands targeting receptors in the ovary rather than in the uterus. 7 receptors were identified in endometrial cancer – AXL, DDR2, TLR4, PTPRF, ITGA2, ITGA3 and ITGB4 –, but only the latter four were expressed on epithelial cells (source of EC).

**Figure 2:**
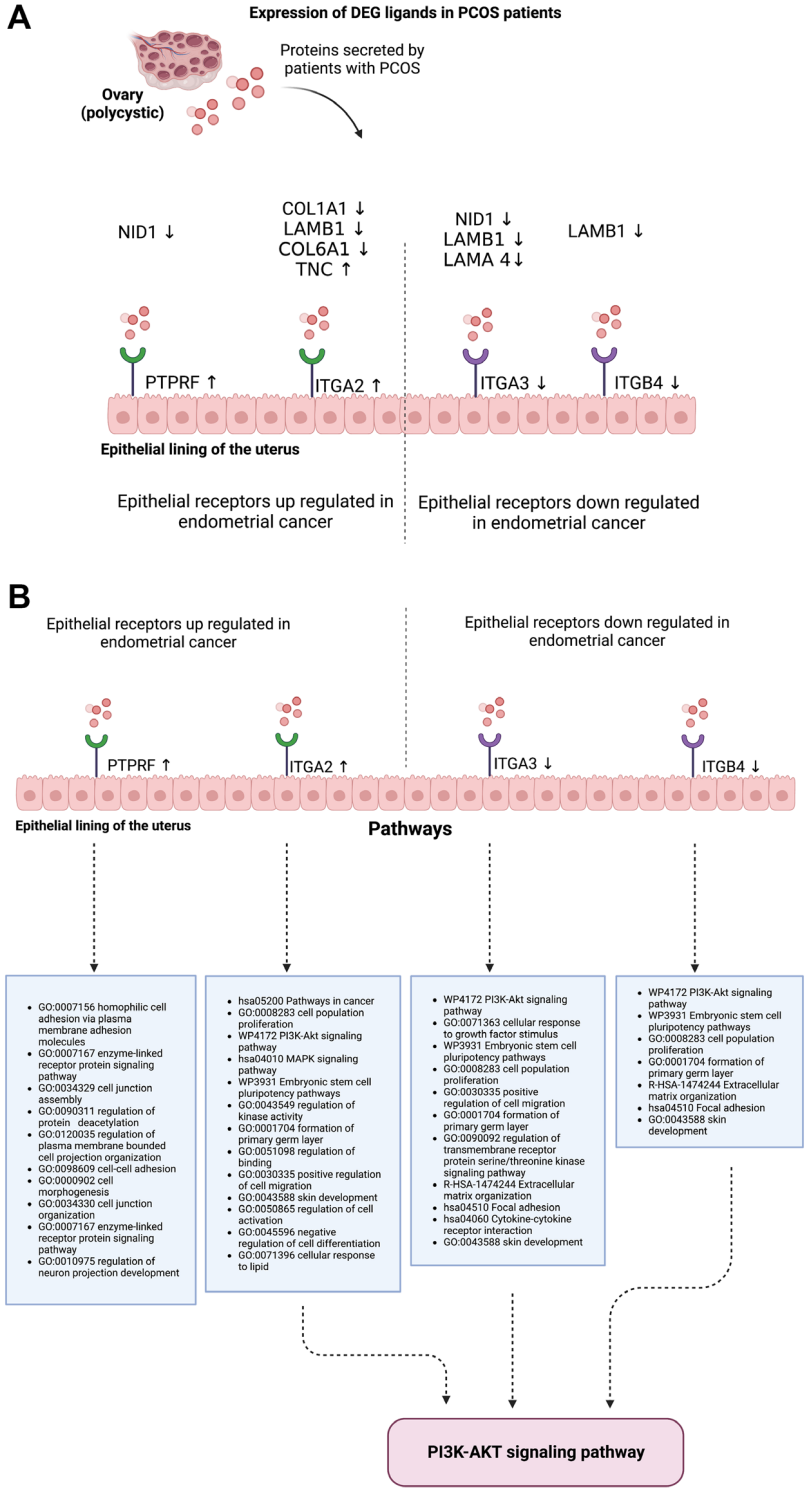
(**A**) The link between PCOS ligands that are differentially expressed and the target receptors in endometrial cancer that are differentially expressed. (**B**) Pathway enrichment of the target receptors in endometrial cancer.

Most of the ligands in PCOS were down regulated in patients, with receptors being either up regulated or down regulated in EC (Figure 2A). Pathway enrichment of these genes show that the major and common pathway involved was that of the PI3K-AKT signalling pathway (Figure 2B). Other pathways related to these receptors include processes involving cellular adhesion and proliferation.

### Other findings on the association between PCOS and EC

#### IGF1 is down regulated in epithelial cells of the endometrium of polycystic ovary syndrome patients

**Figure 3:**
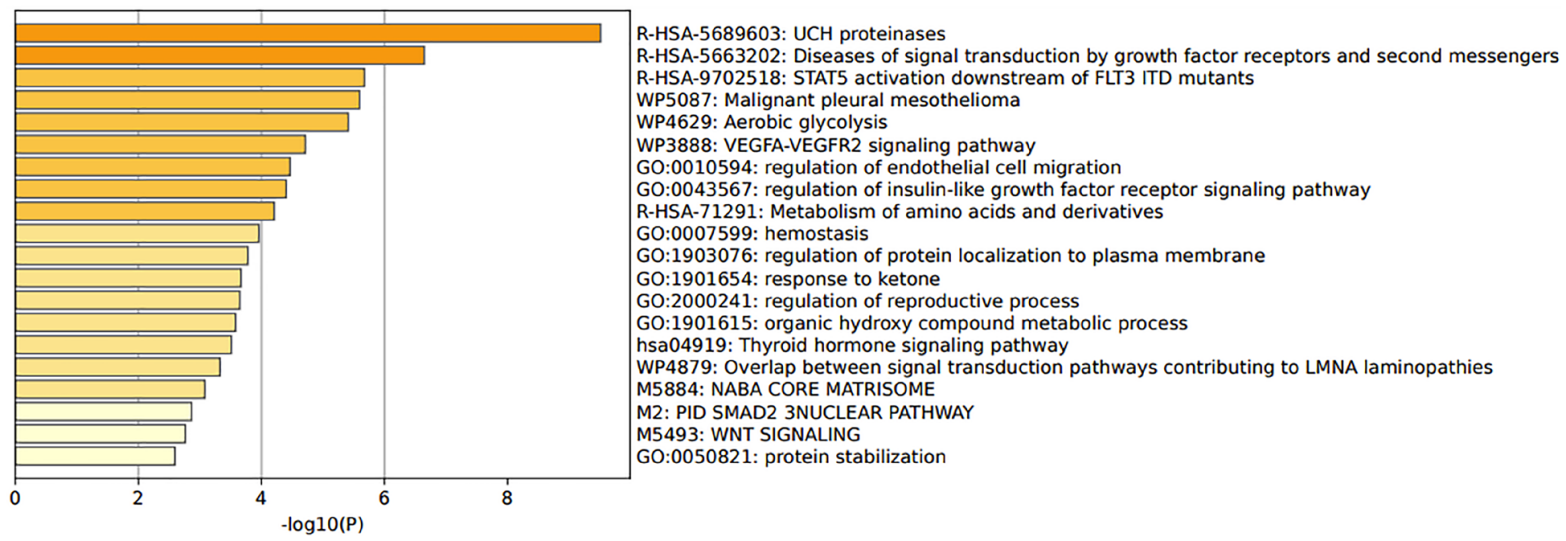
Pathway enrichment analysis of endometrium cells from polycystic ovary syndrome patients in the study GSE48301.

GSE48301 [15] looked at different endometrial cell types of PCOS and healthy women. The reanalysis of data showed that 3 DEGs were considered statistically significant. These genes are IGF1, ALDH1A1, MGP, which have different roles such as being a growth factor or are involved in alcohol metabolic pathways (Supplementary File 2 and Table 1 within Supplementary File 2). However, the top 100 genes were taken and analysed for gene enrichment analysis. The findings are shown in Figure 3. UCH proteinases are involved in an important pathway that mediates protein-protein interactions. Another pathway of interest was the STAT5 activation downstream of FLT3 ITD mutants, as STAT5 contributes to oncogenesis and other pathway members involve several PI3K-AKT pathways (Figure 3; Supplementary File 2 and Tables 2 and 3 within Supplementary File 2).

#### Findings which form the basis for a transcriptomic atlas to underpin future research into PCOS

Table 1 outlines the findings which support the formation of a PCOS transcriptomic atlas. We also found DEGs in common between GSE8157 and GSE6798. There were 54 DEGs in common in skeletal muscle of polycystic patients (Supplementary Table 5), of which 44 were consistently up regulated and 7 were down regulated. However, it is of interest that Lipoprotein Lipase (LPL) and the insulin receptor (INSR) are both down regulated in skeletal muscle. The combined effect of these could explain the observed elevated serum levels of triglycerides found in polycystic patients.

**Table 1:** Findings which form the basis for a PCOS transcriptomic atlas

Study	Methods	Findings
GSE106724 [16] (Supplementary File 3, and Supplementary Tables 3 and 4).	Investigated the long noncoding RNA (lncRNA) and mRNA of ovarian granulosa cells of PCOS patients with either normal testosterone levels or hyperandrogenism in comparison to control patients.	Data reveal that the lncRNA ‘AC142528.1-001’ is up regulated in all types of PCOS and there are 24 down regulated lncRNA’s in all conditions. Furthermore, the one lncRNA that is common between hyperandrogenism, and normal testosterone is ‘LOC100506990’ which is down regulated in both.
(GSE98595) [17] (Supplementary File 4, and Supplementary Figure 1).	Investigated Cabergoline-treated lutein granulosa cells (GSE98595) [17], however only data of non-treated controls and polycystic ovarian syndrome patients were selected in our analysis.	Our reanalysis identified 111 probes as statistically significant DEGs, of which only 75 were assigned to genes. Of these DEGs, 54 were up regulated in PCOS and 21 were down regulated (Supplementary File 4 Table 1). Enrichment analysis of these genes show that most genes are involved in activation of GTPase activity followed by other signalling and receptor pathways such as response to growth factor stimulus.
(GSE1615) [18] (Supplementary File 5, and Supplementary Figure 2).	We reanalysed the data of two different microarray chips to identify the DEGs of theca cells in polycystic patients.	Only two genes were common between the two platforms – MCL1 and NRP2. Gene enrichment analysis for the first platform (U133A) revealed that most DEGs are involved with cell adhesion, RNA splicing and response to growth factors (Supplementary File 5 and Tables 2 and 3, within Supplementary File 5).
GSE40400 [19] (Supplementary File 6, and Supplementary Figure 3).	Investigated human cumulus cells that has been isolated from oocytes at different stages – Metaphase I and Metaphase II – in PCOS patients.	Only two genes were statistically significant, TMED2 and NAB1, both of which were up regulated in the MII stage. Gene enrichment analysis show that most of these genes are involved in embryo development ending in birth or egg hatching, followed by respiratory tube development.
(GSE8157) [20] (Supplementary File 7, and Supplementary Figure 4).	Investigated the effect of effect of pioglitazone on skeletal muscle of insulin resistant PCOS women. Our analysis looked at differentially expressed genes of control subjects with PCOS women prior to treatment.	Gene enrichment analysis showed that most DEGs appeared to be involved in pathways that respond to hormone stimulation.
GSE6798 [21] (Supplementary File 8, and Supplementary Figure 5).	Investigated skeletal muscle of obese PCOS women and control patients	Of the down regulated genes, the insulin receptor (INSR), appeared to be involved in six of the ten enriched pathways, positive regulation of ryanodine-sensitive calcium-release channel activity; sensory perception of temperature stimulus; distal deletion syndrome; Neuroinflammation and glutamatergic signalling; Longevity regulating pathway; (Supplementary Figure 5).

#### Findings which form the basis for a transcriptomic atlas to underpin future research into endometrial cancer

Table 2 outlines the findings which support the formation of an EC transcriptomic atlas. While most of the endometrial cancer studies had different inclusion criteria, all studies included stage I tumours derived from endometroid cells. All DEGs were intersected to identify commonalities (Supplementary Figures 6, 7 and Supplementary Table 6) and GSE 115810 [22] was the only dataset to share some genes with some of the other datasets identified. However, the expression of these genes was different between each dataset (Supplementary File 1 and Supplementary Tables 1–3). We identified 12 genes that were shared between endometrial cancer of different tumour grades and in deceased patients. Of those genes, only 1 (CORO1B) was commonly up regulated in all datasets, 10 were commonly down regulated and one gene up regulated in one data set and down regulated in the other.

**Table 2:** Findings which could form the basis for an EC transcriptomic atlas

Study	Methods	Findings
(GSE115810) [22] (Supplementary File 9, and Supplementary Figure 8).	Investigated expression between different grades of endometrial cancer. We analysed the data between healthy controls and patients.	10 genes that were differentially expressed in endometrial cancer, regardless of the tumour grade. Gene enrichment analysis showed there were several pathways involving cellular adhesion.
GSE17025 [23] (Supplementary File 10, and Supplementary Table 7 and Supplementary Figure 9).	Investigated the effect of different stage I endometrial cancers in patients with different histological subtypes. The top 100 DEGs were considered as only 40 genes were found to be statistically significant.	Gene enrichment for all DEGs showed that microtubule-based movement was the most affected by these genes.
(GSE178671) [24] (Supplementary File 11, and Supplementary Table 8 and Supplementary Figure 10).	Investigated endometroid cells from endometrial cancer in patients with recurrent and non-recurrent cancer. Our reanalysis of the data was based on those who are recurrent against those who are non-recurrent.	Two genes, FN1 and DKK1, were identified to have been statistically significant. FN1 encodes fibronectin 1, which is involved in cell adhesion and migration, while DKK1 plays a role in Wnt signalling and embryonic development. Pathway enrichment analysis showed a high involvement of several oncogenic pathways
GSE21882 [25] (Supplementary File 12, and Supplementary Tables 9 and Supplementary Figure 11).	Investigated stage I endometrial tumours of survivor and non-survivor patients. In our reanalysis, we analysed mortality regardless of external factors and comorbidities, as the number of samples for many of these factors fell under the threshold needed for an analysis to be carried out.	Gene enrichment analysis via Metascape showed that most genes were linked with pathways responsible for the regulation of nuclear beta catenin signalling and target gene transcription, and negative regulation of cell population proliferation.

## DISCUSSION

The aim of this study was to investigate the possible molecular associations between PCOS and EC by analysing data from publicly available transcriptomic data sets. We set out to further investigate findings from previous research showing that IGF-1 and IGFBP gene expression in the endometrium of women with PCOS and EC was increased compared with normal endometrium and identify other novel genes that may be commonly expressed. We were unable to confirm these specific findings from our data analysis at this stage, especially as this was an *in-silico* analysis and laboratory validation is required to support any hypothesis that can be generated from this analysis. Both IGF1 and INSR were however down regulated in PCOS and EC patients, when we looked at the DEGs that were common between all PCOS datasets and all EC datasets, except for one dataset (GSE178671) where it was up regulated in recurrent endometrial cancer stage IB in comparison to non-recurrent endometrial cancer stage IB. This finding goes against the findings from the pilot study in the UK [14] where IGF1, IGFBP1 and PTEN gene expression were significantly up regulated in the endometrium of PCOS and EC women compared to controls, so more studies are required to decipher the exact role of the insulin signalling pathway in EC risk in PCOS.

On the other hand, several of the DEGs were related to insulin and insulin-related pathways such as IGFALS, IGFB5, SYNAP5, IGF2BP2-AS1, and IGFBP4. Pathways affected by or involving insulin include GO:1990314 cellular response to insulin-like growth factor stimulus, hsa04910 Insulin signalling pathway, hsa04931 insulin resistance. It is of interest that in the study GSE178671, where recurrent and non-recurrent endometrial cancer were compared, the DEGs were involved in the IGFR1 cascade signalling, signalling by type 1 insulin-like growth factor-1 receptor, IRS-related events triggered by IGF1R, all of which are within the MAPK signalling pathway (Supplementary File 11 and Table 3 within Supplementary File 11). The MAPK signalling pathway when exposed to growth factors stimulates the cells to proliferate and grow and is a known tumour suppressor pathway. Changes in the insulin signalling pathway may therefore underpin the association between PCOS and EC as insulin resistance is a key feature of PCOS, though the precise molecular mechanisms which underpin this association remains unclear.

Despite this uncertainty, we believe our work has identified some novel findings and provide sufficient data to form the basis for a transcriptomic atlas to improve our understanding of the underlying mechanisms as well as underpin future research into PCOS and EC. Similar transcriptomic atlases have been published for other disease states e.g., COVID-19 [26]. For example, one interesting finding was the identification of four receptors present on epithelial cells of endometrial cancer, potentially linking EC with PCOS. To find out how PCOS may be promoting EC, one must first find out what PCOS is putting into the blood stream that is not found in the same amounts as in healthy blood. Ideally this should be metabolites as well as excreted gene products (proteins and possibly RNAs).

From our transcriptomic atlas, the focus was on extracellular proteins. Using the human protein atlas, we successfully identified which DEGs encoded proteins secreted in the blood, and their receptors on the epithelial lining of the uterus were identified using the online tool, EcoTyper. Four receptors (PTPRF, ITGA2, ITGA3 and ITGB4) were identified as differentially expressed in EC with ligands or gene products secreted by PCOS. While these receptors appeared to be either up or down regulated in endometrial cancer, their ligands appear to be mostly down regulated by PCOS.

Next, it was important to determine which of these molecules were known to be signalling molecules and what their effects were in terms of altering gene expression or enzyme activity (i.e., compile an effector list). Following this, it was important to determine which gene expression changes in EC were linked to a cancerous state. In terms of the identified receptors, three – ITGA2, ITGA3 and ITGB4 – appeared to be involved in the PI3K-AKT signalling pathway, which is a known oncogenic pathway that promotes various cell functions such as proliferation, cell survival and angiogenesis. PTPRF, the fourth gene, was an up regulated receptor gene in EC and involved in various pathways linked to cellular adhesion and organization. Its ligand, NID1, a gene that plays a role in cellular adhesion and extracellular matrix organization, appeared to be down regulated in PCOS. Subsequent research to decipher the precise mechanisms by which these receptors might play a role in the association between EC and PCOS should ideally find out from the literature what molecules cause these gene expression changes, and in a pathway analysis, these can be linked with their target affected genes.

The results of this study also provided data to form the basis for a transcriptomic atlas to underpin future research into the molecular mechanisms underpinning PCOS and/or EC. For example, in PCOS, we found that in the study GSE106724 [16] that looked at the long noncoding RNA (lncRNA) and mRNA of ovarian granulosa cells of PCOS patients with either normal testosterone levels or hyperandrogenism, in comparison to control patients, the lncRNA, ‘AC142528.1-001’, was up regulated in all conditions of PCOS regardless of testosterone and androgen levels, and the lncRNA ‘LOC100506990’ was down regulated in both PCOS patients with hyperandrogenism and normal testosterone levels. This could form the basis for a novel PCOS biomarker and further illustrates the value in publishing our transcriptomic atlas at this stage.

Other findings were the association between DEGs involved in activation of GTPase activity receptor and pathways, such as response to growth factor stimulus and lutein granulosa cells in PCOS (GSE98595) [17]; pathways associated with cell adhesion, RNA splicing and response to growth factors in PCOS theca cells (GSE1615) [18]; embryo development ending in birth or egg hatching, followed by respiratory tube development and oocytes in Metaphase II in PCOS patients GSE40400 [19]; response to hormone stimulation (including the response to insulin pathway) in skeletal muscle of insulin resistant PCOS women (GSE8157) [20], the finding of a pathway involved in positive regulation of ryanodine-sensitive calcium-release channel activity in DEGs identified in the skeletal muscle of obese PCOS women GSE6798 [21] and the down regulation of LPL gene in skeletal muscle in PCOS patients (GSE6798).

Furthermore, when we investigated the pathways associated with the 54 DEGs in skeletal muscle of PCOS patients from two different studies (GSE8157 [20] and GSE6798 [21]), gene enrichment of the up regulated genes revealed that the genes were involved in: (1) activation of RAC1 downstream of NMDARs, (2) behaviour and (3) multicellular organismal homeostasis. Of these three signalling pathways, RAC1 has been found to be hyperactivated in human cancers and promotes tumour initiation, progression, and metastatic dissemination [27], and may be consistent with a link between PCOS and EC.

Findings which form the basis for a transcriptomic atlas to underpin future research into EC included the association between cellular adhesion and grades 2 and 3 EC (GSE115810) [22], microtubule-based movement in stage 1 EC (GSE17025 [26], an association between cell adhesion and migration and Wnt signalling with recurrent endometrial cancer (GSE178671) [24], the regulation of nuclear beta catenin signalling and EC survival (GSE21882) [25] and the identification of CORO1B gene as the only up regulated gene common to all datasets of EC of different tumour grades and in deceased patients, which may be involved in cell motility.

The use of gene-enrichment analysis (GEA) to identify potential pathways was a strength and helpful step to enable us to investigate our research question. We are also unaware of a similar approach to decipher the possible molecular links between PCOS and EC. A potential limitation however is that results from GEA can sometimes be misleading. The differences identified may reflect the fact that each disease is caused by different factors (input signals) and DEG changes in the same direction might be coincidental or due to some other factor exerting the same change in both diseases. The same change in gene expression in both diseases might not have anything to do with one (PCOS) causing a change in the other (EC). Having identified a potential pathway, it is then crucial to look at what is happening to the expression levels of all the components. Several members of a pathway might have elevated transcript levels while others might have reduced levels or have elevated levels of inhibitory factors which nullify the effectiveness of the pathway. Further interrogation of the data presented in our transcriptomic atlas is therefore required.

Our transcriptomic atlas allows research groups globally to independently undertake further data analyses to investigate the molecular links between PCOS and EC. We identified four novel receptors which may underpin the risk of EC in PCOS, but there may well be others. Finally, both IGF1 and INSR were down regulated in PCOS and EC patients, which was not consistent with previous research. Therefore, more studies are required to decipher the exact role of the insulin signalling pathway in EC risk in PCOS.

## MATERIALS AND METHODS

Institutional review board approval was not required for this study as we have used publicly available data, and the study was conducted between February and May 2022. The Gene Expression Omnibus (GEO) database from NCBI was used to identify transcriptomic studies containing the keywords ‘polycystic ovary’ and ‘Endometrial cancer’ [28]. Of the identified studies, only datasets containing expression profiling by array, and gene expression profiling by high throughput sequencing were selected for analysis.

Differentially expressed genes (DEGs) were identified using the built-in GEO2R program with the limma (Linear Models for Micorarray Analysis) R package and applying forced normalization on all data. Comparisons used for analysis were between healthy controls and cases, or different grades of tumours (Supplementary Figure 12 and Supplementary Table 10). Genes with an adjusted *P* value of ≤ 0.05 were considered as statistically significant. All DEGs were analysed using the Metascape database, a gene enrichment and analysis resource, for identification of pathways [29, 30].

**Figure 4:**
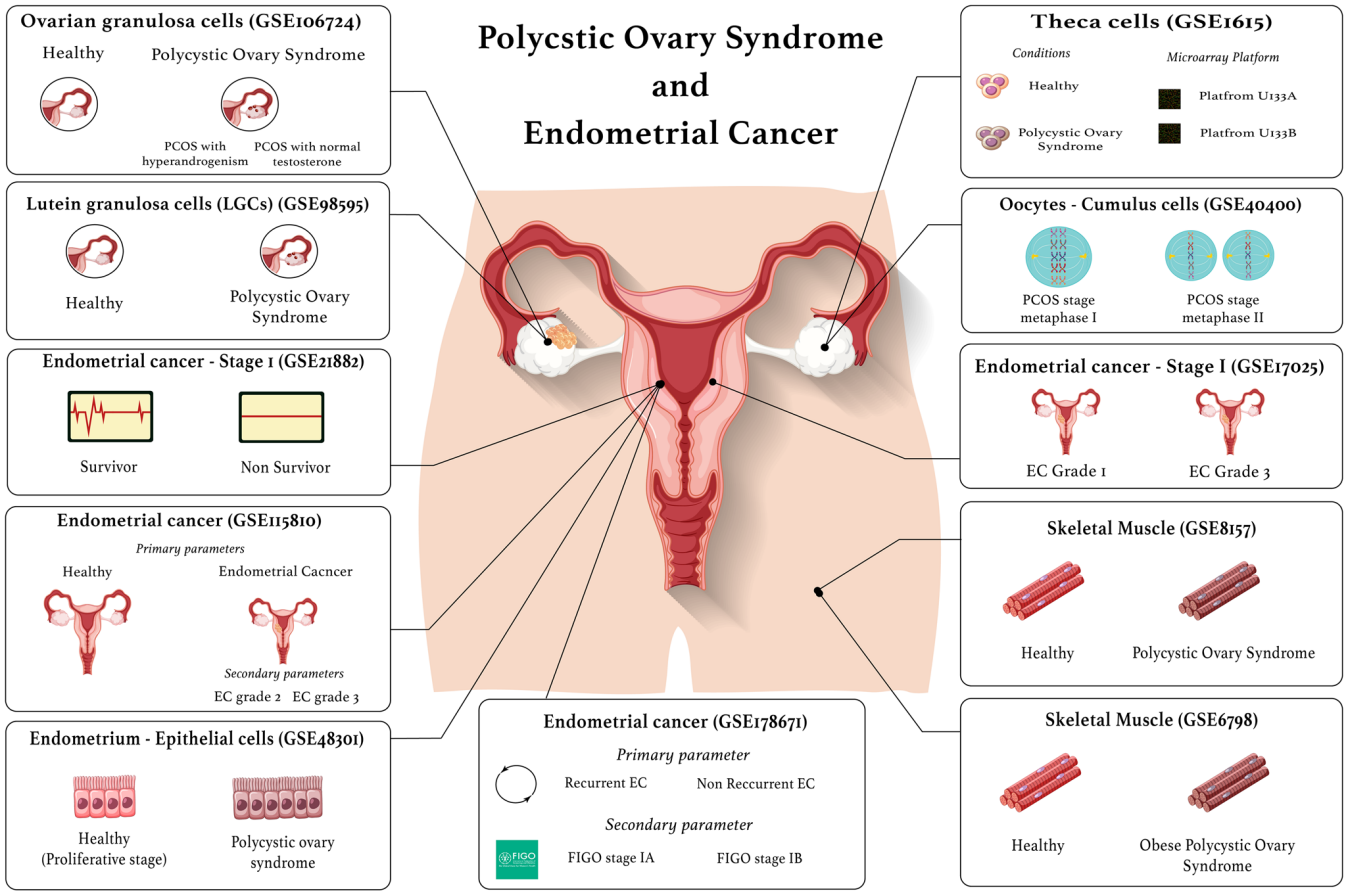
Visual representation of the datasets used in this study.

PCOS DEGs encoding proteins that are secreted in the blood were identified using the Human Protein Atlas blood protein database [31]. The endometrial cancer DEGs that are epithelial receptors were identified using EcoTyper [32]. Furthermore, this tool was used to identify the receptors for the blood protein encoding PCOS genes, and the ligands for the EC receptors. An up-set plot was generated using R studio (R version 4.2.1) to identify the common secreted ligands and common receptors in PCOS and endometrial cancer. Figure 4 is a visual representation of the datasets used in this study.

## SUPPLEMENTARY MATERIALS



























## Abbreviations

PCOS: Polycystic Ovary Syndrome
EC: Endometrial Cancer
DEGs: Differentially expressed genes
NCBI: National Center for Biotechnology Information
GEO: Gene Expression Omnibus
LncRNA: long noncoding RNA
mRNA: messenger RNA
