# Supplementary material for: Transcriptomic analysis identifies four novel receptors potentially linking endometrial cancer with polycystic ovary syndrome and generates a transcriptomic atlas
Source: Oncotarget. 2023 Sep 22;14:825–35. doi: 10.18632/oncotarget.28513 (PMC10515731; doi:10.18632/oncotarget.28513)
Supplement: Supplementary file 1 [file oncotarget-14-28513-s001.pdf]

Transcriptomic analysis identifies four novel receptors potentially linking endometrial cancer with polycystic ovary syndrome and generates a transcriptomic atlas

SUPPLEMENTARY MATERIALS

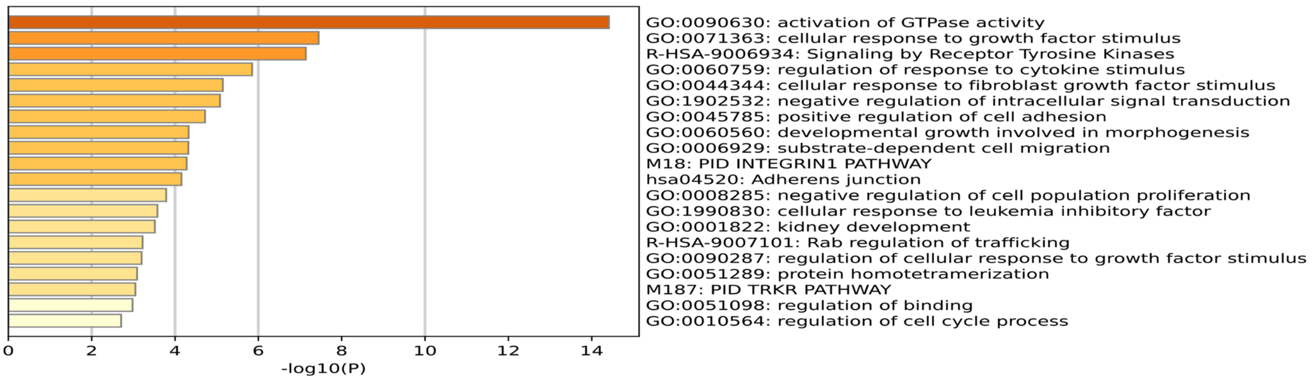

Supplementary Figure 1: Gene enrichment of differentially expressed genes in granulosa cells of PCOS patients from the study GSE98595 using metascape.

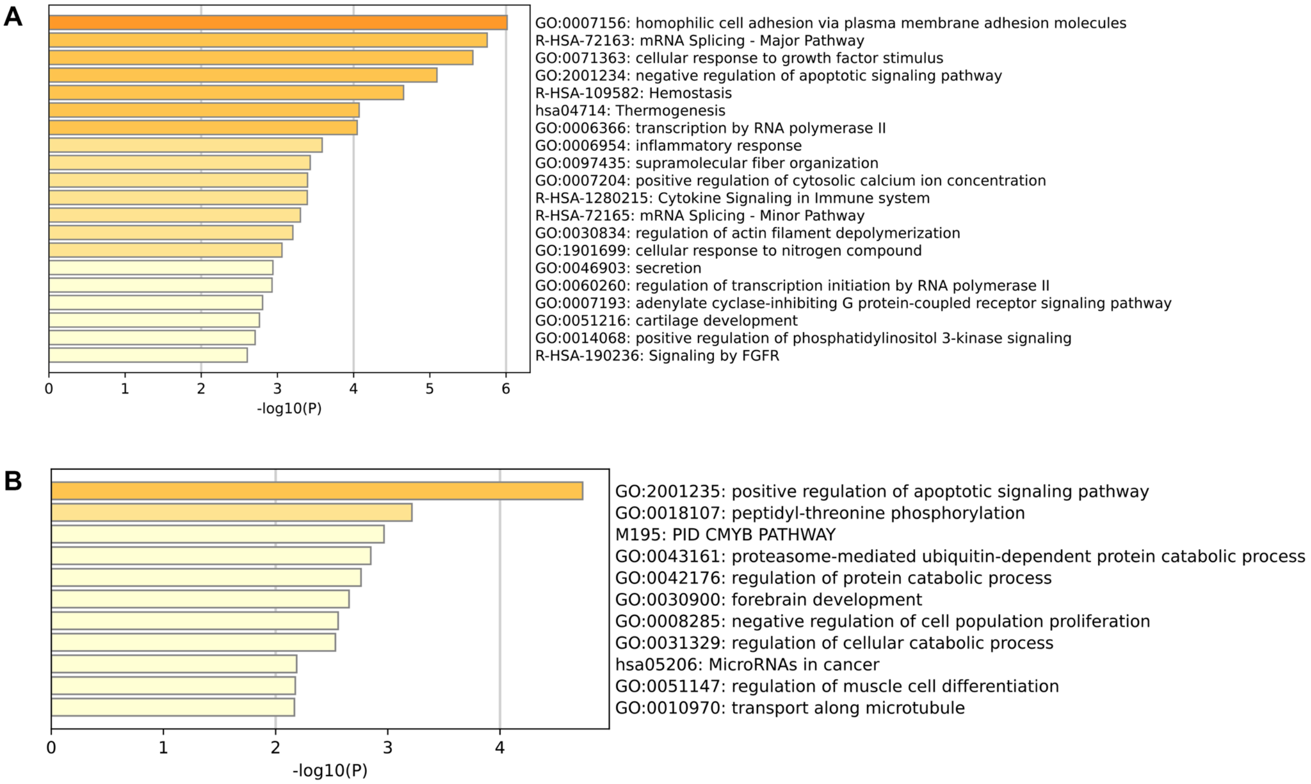

Supplementary Figure 2: Gene enrichment analysis of DEGs identified in GSE1615 using the (**A**) Affymetrix Human Genome U133A Array, and the (**B**) DEGs identified using the Affymetrix Human Genome U133B Array using metascape.

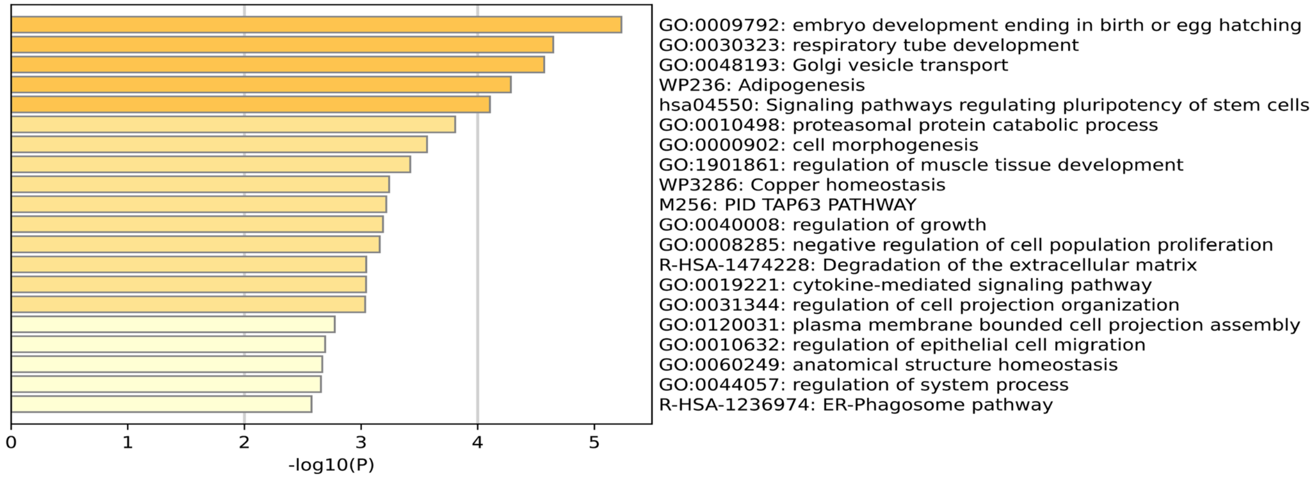

Supplementary Figure 3: Gene enrichment analysis of oocytes in metaphase II stage in PCOS patients in GSE40400 using metascape.

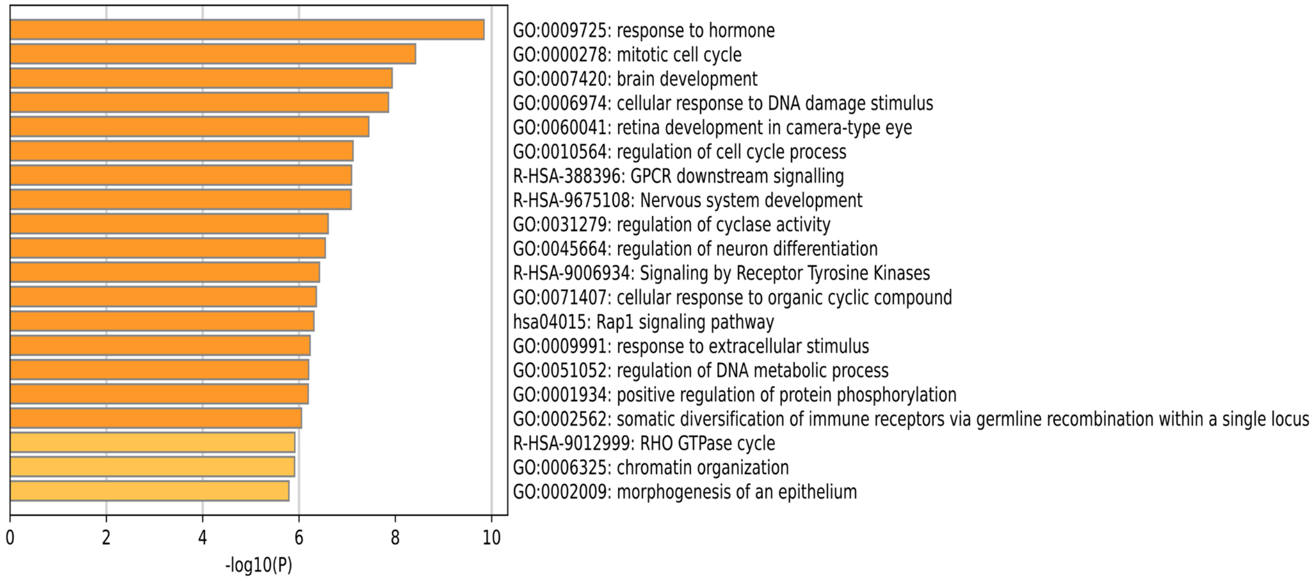

Supplementary Figure 4: Gene term enrichment of differentially expressed genes in skeletal muscle of polycystic patients in the study GSE8157 using metascape.

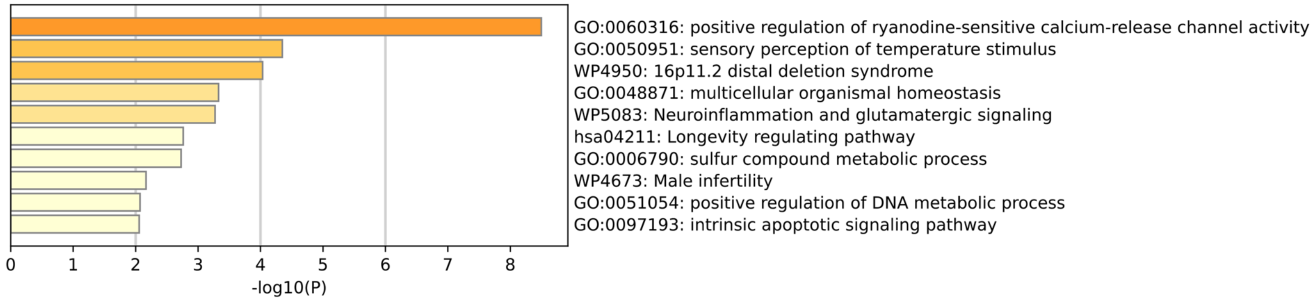

Supplementary Figure 5: Gene enrichment differentially expressed genes in skeletal muscle of obese polycystic women from the study GSE6798 using metascape.

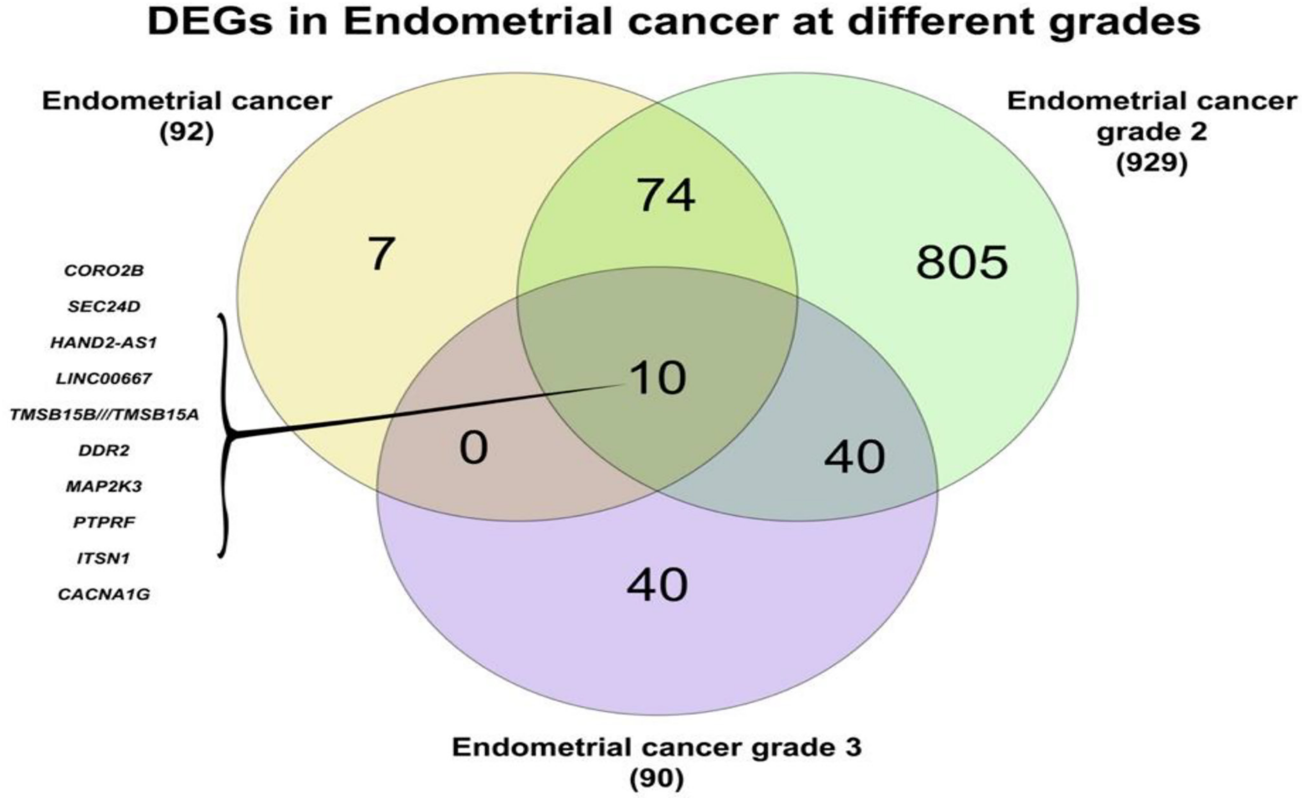

Supplementary Figure 6: DEGs for endometrial cancer from the dataset GSE115810.

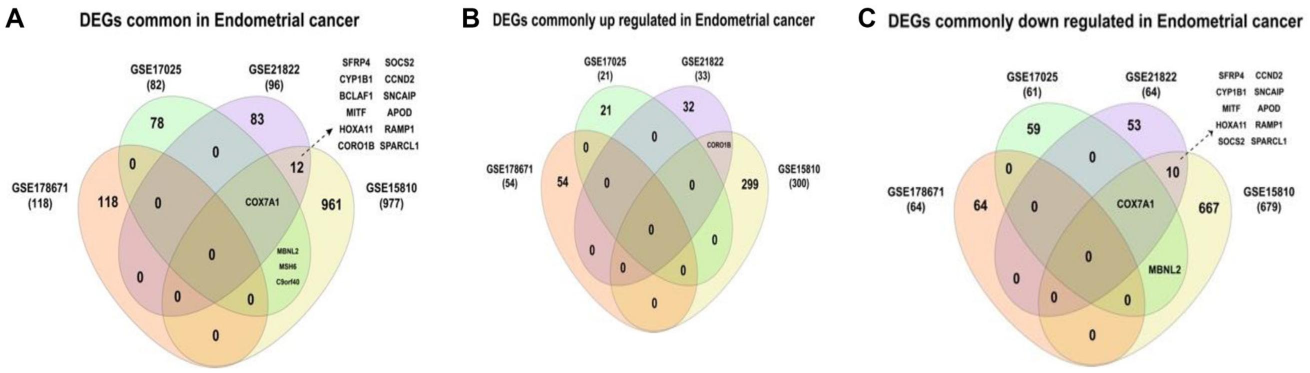

Supplementary Figure 7: (**A**) all common DEGS between all data sets and (**B**) common up regulated DEGS in all datasets and (**C**), the common down regulated genes.

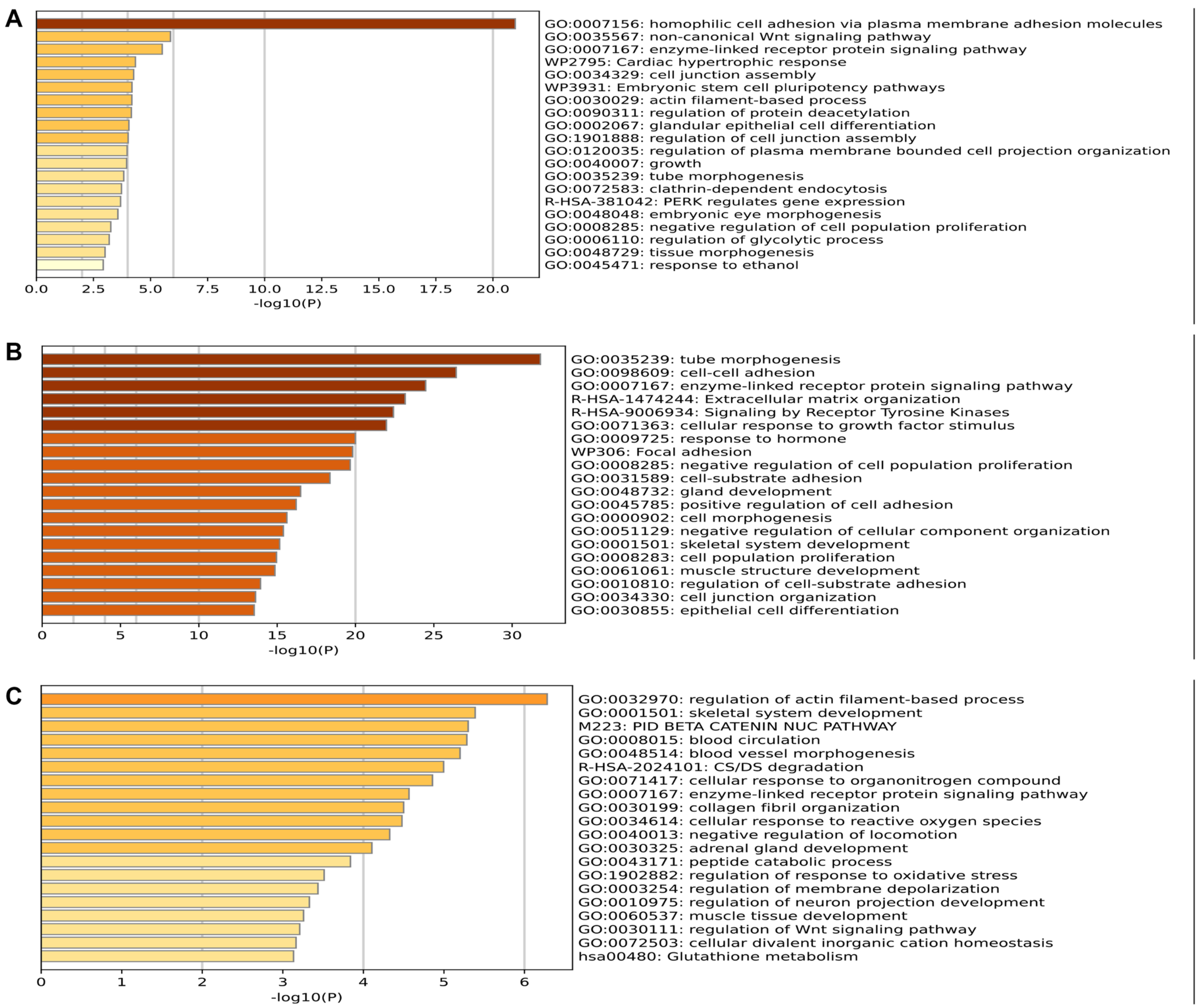

Supplementary Figure 8: Gene enrichment analysis of GSE115810 based on (**A**) endometrial cancer, (**B**) grade 2 endometrial cancer and (**C**) grade 3 endometrial cancer using metascape.

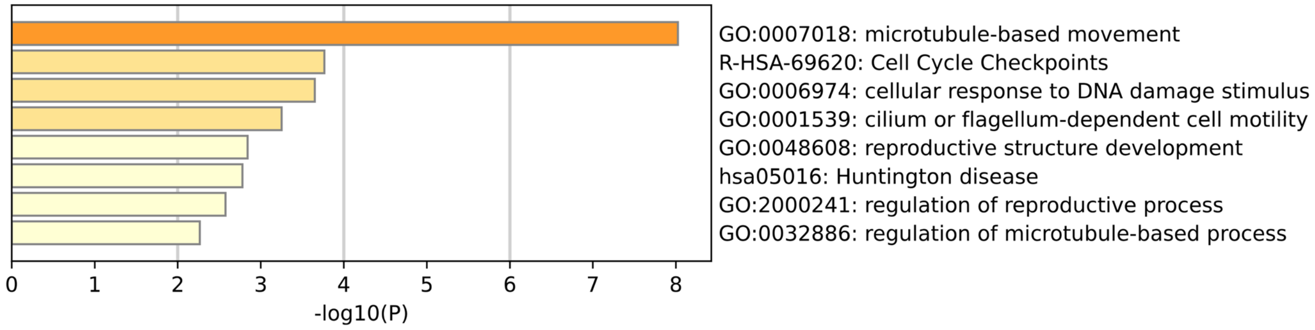

Supplementary Figure 9: Gene enrichment analysis using metascape for DEGs of endometroid cells obtained from stage I grade 3 endometrial cancer patients from the dataset GSE17025.

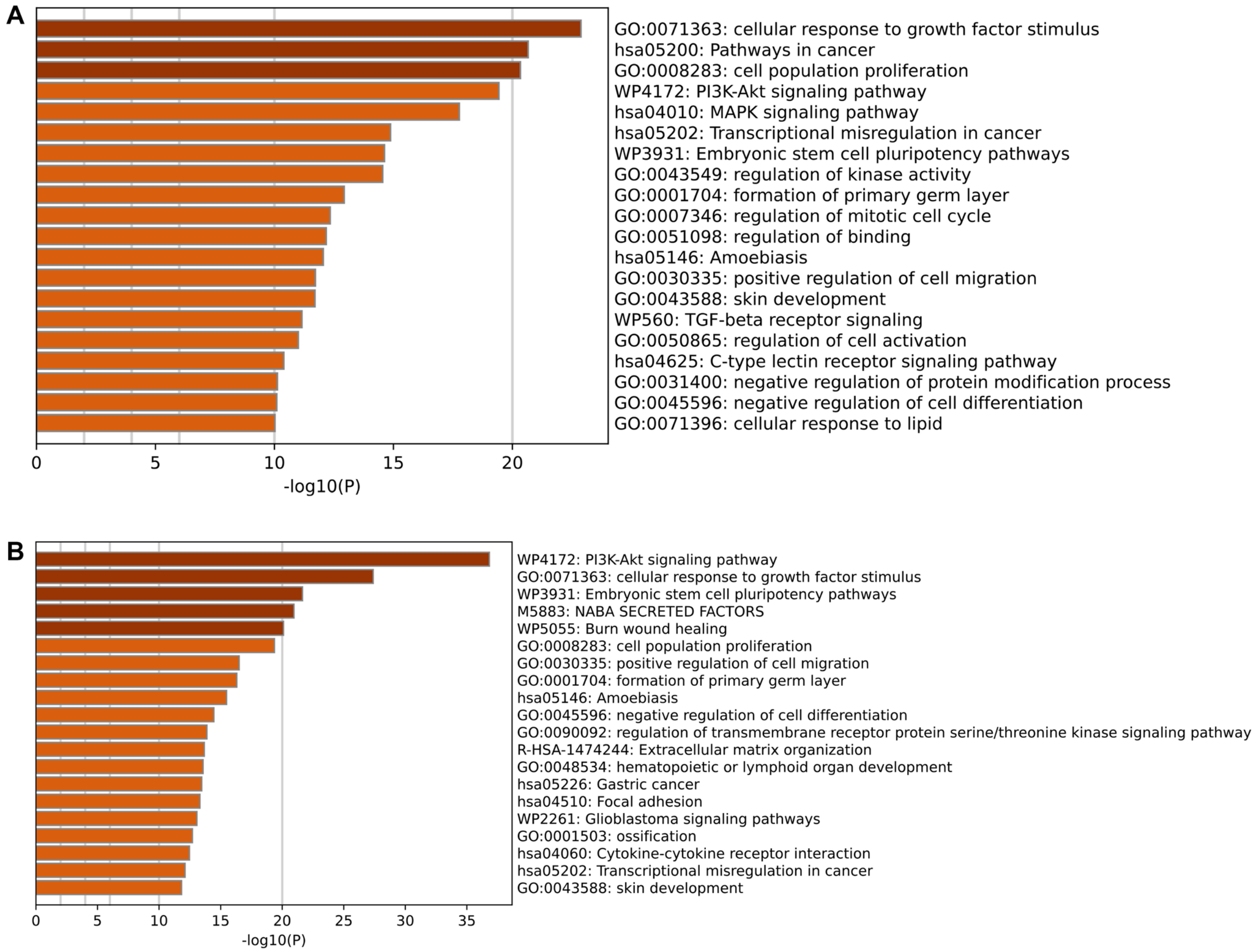

Supplementary Figure 10: Gene enrichment analysis of genes (**A**) DEGs in recurrent endometrial cancer, and (**B**) DEGs in recurrent stage IB endometrial cancer from the study GSE178671 using metascape.

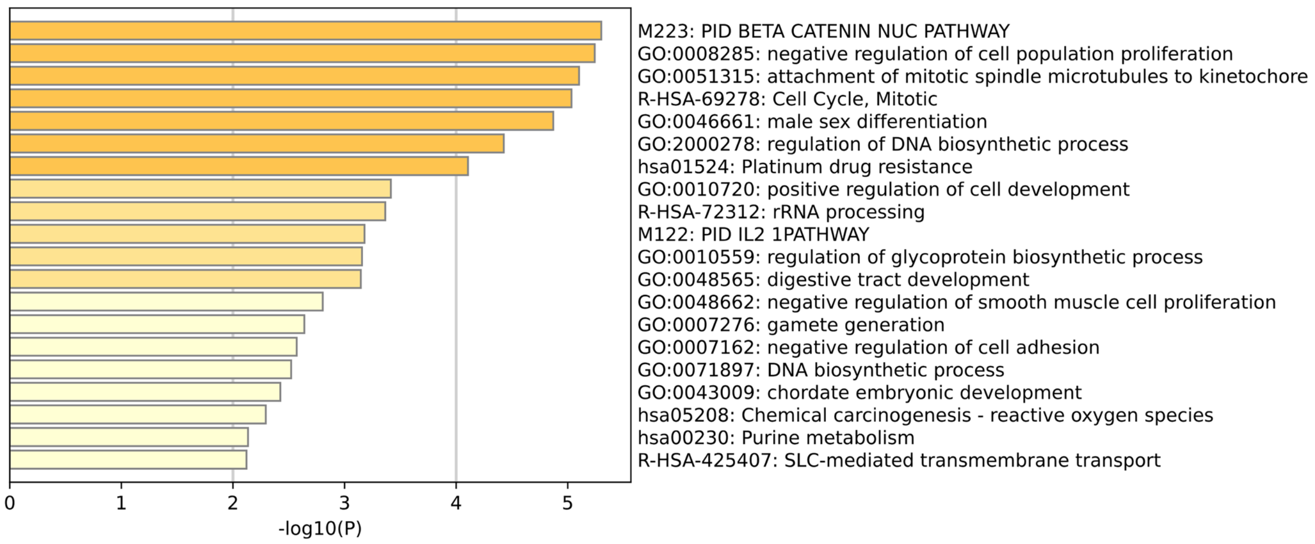

Supplementary Figure 11: Gene enrichment analysis of DEGs of non survivors of stage I endometrial cancer in GSE21882 using metascape.

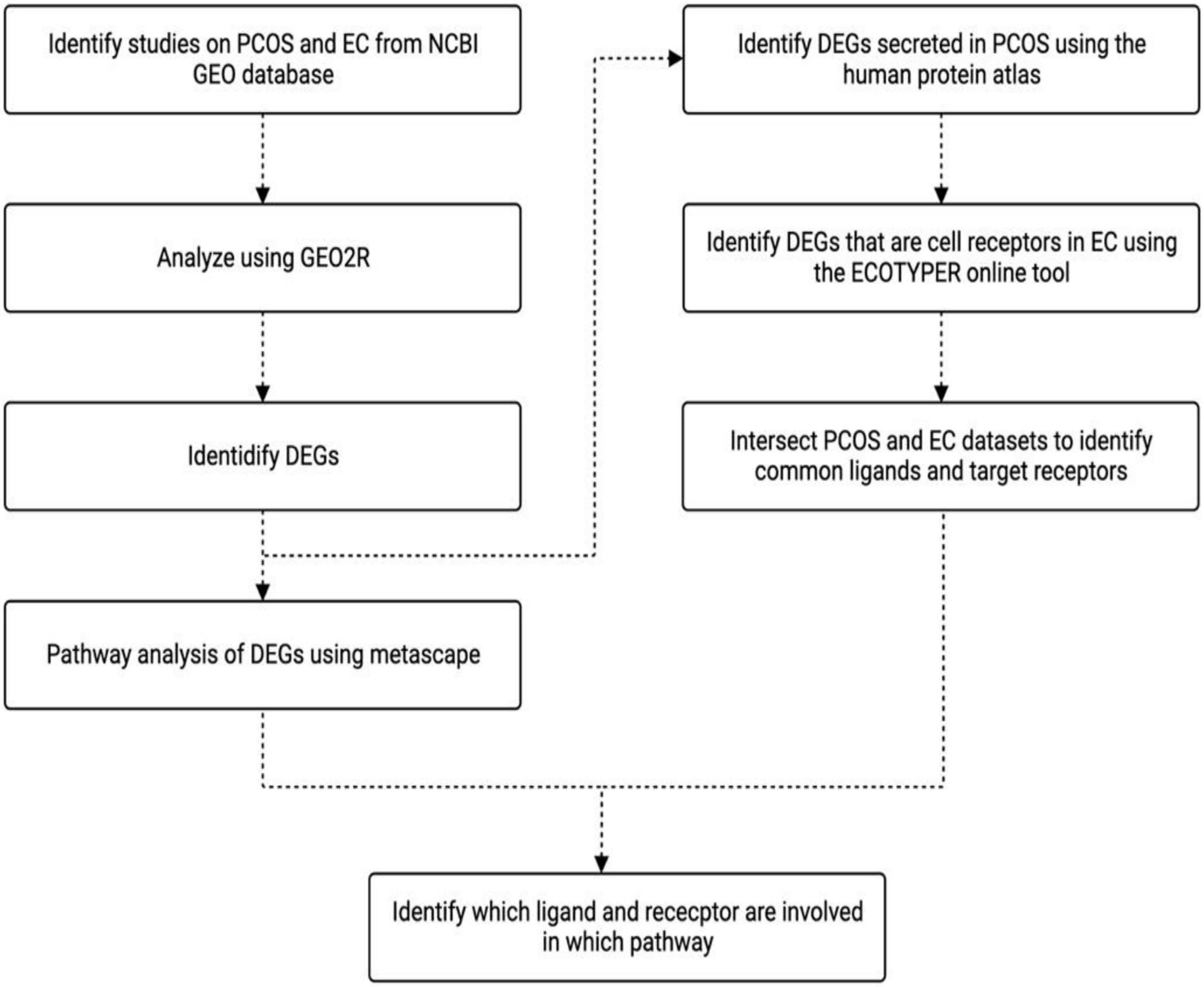

Supplementary Figure 12: Chart depicting the workflow process for the analysis carried out in this study.

Supplementary Table 1: List of PCOS DEGs that are secreted ligands

Cell source	Study	Protein coding genes (Ligands)
Granulosa cells	GSE106724	TMEM87A, ITIH1, IGFALS, P4HA2, CMA1
	GSE106724 – PCOS with hyperandrogenism	FIBIN, TNC, TNN, LPL, VSTM4, CRHBP, RNASET2, GDF3, TNXB, KREMEN2, GPC5, MDK, ADAMTS17, TMEM87A, LAMB1, PRSS21, FSHR, CSGALNACT1, GNLY, RAET1E, PRB3, IGFALS, NDNF, NELL2, AGRN, FCN3, XYLT1, BCHE, LCN12, B3GNT7, PRSS3, SFRP4, ITIH1, ISM1, MATN3, CHST1, OLFM1, CMA1
	GSE98595	HSPA1A, CD24, LAMA4, SLIT2, COL6A1, SERPING1, FABP6, SYAP1, THBS2, RTN4RL1, NGRN, FSTL3, NID1
	GSE106724 – PCOS with normal testosterone levels	ZPLD1, TMEM87A
Theca cells	GSE1615 – Platform 1 (U133A)	PCDHGA11, HMGB1, CD44, FLRT2, HNRNPA2B1, LTBP4, BGN, COL1A1, MAZ, GNS, ADAM28, NRP2
	GSE1615 – Platform 2 (U133B)	NRP2, EMCN, BMP6
Oocyte	GSE40400	COL9A1, RCN3, C1S, ANGPTL1, IL17RB, LIFR, ADAM10, SFRP4, ADAMTS9, IGFBP5, IFNLR1, OSMR
Endometrium	GSE48301	IGF1, MGP, GDF9, COL14A1, F13A1, PAMR1, SFRP4, SECISBP2, PDGFRA, C1orf54, LYG2, OGN, IGFBP5, SPARC, C1S
Skeletal muscle	GSE8157	GYPB, LNX1, ANGPTL1, MUC5B, HMCN2, MSH3, XYLT2, BCKDHB, FUT6, NXPE1, ADAMTS5, LAMB3, CELA3B, COCH, OXT, CALCA, FGF22, NLRP3, PGC, HS6ST1, ALG1, ASCL1, ACP6, C11orf94, FUT10, MUC2, ADAMTS8, LECT2, NCAM1, PRDM12, IGKC, PDZD8, NUCB1, CSH1, CELSR1, ILDR1, NID1, MDGA1, FGB, HMGB2, RNF150, COL5A1, CTRB2, ZBED3, LAMA4, IFNW1, CTSA, GPC1, LOX, CCK, CRB2, COL6A1, TFPI, VSTM2A, COLGALT2, ADAMTSL1, TNFRSF25, RARRES1, CFI, C17orf67, PON2, CDSN, HSD11B1L, ADGRG1, ITFG1, CD24, GFRA2, CERCAM, FREM1, PRELP, SVEP1, GUCA2B, SPP1, A2M, GFOD2, SFN, STOX1, EMILIN2, LAMA1, ACVRL1, NECTIN1, RDH12, NTN4, CFAP65, FGFR4, ACACB, OBP2B, DMBT1, DNASE1, TFRC, CCL19, SMOC1, PGLYRP1, DMP1, NECAB3, C14orf93, MFAP4, OSM, PSAP, MSLN, ST6GAL1, SECISBP2, WFIKKN1, GGA2, ITGB3, IPO9, CXCL2, HS6ST3, IGLV1-44, GHRHR, GHRL, LPL, IL6, GP2, SIAE, MCF2L, HHIPL2, ANGPTL8, CPXM2, IL2, CNOT9, GPLD1, B3GNT8, TGFB3, LAMA3, HPX, PPIB, CD44, CLCA4, ANGPT2, EDIL3, MUC4, FAT1, BTD, C1orf56, B3GALT5, ICOS, EFNA4, MGAT5, FAM20A, SLC25A14, SNTB2, FJX1, KCP, ADAM18, CALU, ELANE, APOE, IL1R2, MCEE, NTN1, CD209, IL17RC, TMEFF2, OAF, SLC8A1, ARSK, SUSD4, PI3, RDH11, CCL24, THBS1, LILRA5, GXYLT1, MELTF, MFAP3L, EDA, OLFM2, FGF7, EMCN, PRSS12, MRPS22, MUC1, CLN5, FGF17, C7, C4BPB, PDHA1, MRPL35, IGHD, THBS2, CRTAP, F7, CSHL1, SCUBE2, GNAS, TPSAB1, IL12RB1, B4GALNT3, PF4, P3H2, MCCC1, DKK3, SBSPON, WNT4, CEP89, PCSK6, PXYLP1, TCN1, EDN3
	GSE6798 – Obese PCOS	PDIA5, RSPO3, GYPB, LPL, ST3GAL2

Supplementary Table 2: Endometrial cancer DEGs that are receptors

Study	Epithelial surface receptor
GSE115810	ROR2, DDR2, PTPRF, EPOR, CDH2
GSE115810 – Grade 2 endometrial cancer	P2RY14, PTPRF, RAMP1, NPR2, EDNRA, PTH1R, PTCH1, AXL
GSE115810 – Grade 3 endometrial cancer	AXL, NRP1, TMEM8A, DDR2, CLEC2B, PTPRF, HTR2B, KIDINS220, NPR2
GSE17025 – Grade 3 endometrial cancer	SLC40A1
GSE178671 – Recurrent endometrial cancer stage IB	MET, FGFR2, CSF3R, FZD7, TNFRSF10D, TNFRSF10C, ITGB8, IL6R, ITGA3, FGFR1, TLR4, HHIP, NOTCH2, ITGB4, PDGFRB, IL1R2, FGFR4, BAMBI
GSE178671 – Recurrent endometrial cancer	MET, FGFR2, ITGA2, PDGFRB, HHIP, LEPR, CD19, BAMBI, TLR4
GSE21882	IL6ST, RAMP1

Supplementary Table 3: Number of differentially expressed lncRNA's identified in the different conditions analysed in GSE106724

Healthy control vs. PCOS	Up regulated in PCOS: 15 lncRNA<br>Down regulated in PCOS: 146 lncRNA
Healthy control vs. PCOS with hyperandrogenism	Up regulated in PCOS: 454 lncRNA<br>Down regulated in PCOS: 397 lncRNA
Healthy control vs. PCOS with normal testosterone levels	Up regulated in PCOS: 6 lncRNA<br>Down regulated in PCOS: 62 lncRNA

Supplementary Table 4: Number of common lncRNA when intersecting different conditions analysed in GSE106724

PCOS	41 lncRNA
PCOS with hyperandrogenism	740 lncRNA
PCOS with normal testosterone levels	32 lncRNA
PCOS and PCOS with hyperandrogenism	85 lncRNA
PCOS and with normal testosterone levels	10 lncRNA
PCOS with hyperandrogenism and PCOS with normal testosterone levels	1 lncRNA
PCOS and PCOS with hyperandrogenism and PCOS with normal testosterone levels	25 lncRNA

Supplementary Table 5: Common differentially expressed genes in skeletal muscle of PCOS using the datasets GSE8157 and GSE6798

Up regulated genes	VSIG1, CD53, VN1R5, MSS51, SMIM11A, PPIE, POLB, CAMKK2, ADAM11, C2orf61, NFYC-AS1, GSE1, NPAS2, LOC100505841, SLC1A2, LOC105373113, CALM3///CALM2///CALM1, RCC1L, SHPK///TRPV1, LAMB2P1, NANOS1, GLG1, MYH4, C2orf68, GYPB, LOC283482, ZXDC, SMARCA4, LOC100653137///CDH23, LOC283887, KCTD7///RABGEF1, ARL5C, MYL10, PAX8, FCER1A, ACTG1P17, OPN4, LOC101927151, ORAI2, CYP3A43, ZDHHC8, MAD1L1, GLRA2, TNPO2
Down regulated genes	INSR, EMC3, BTBD9, LDHB, GSTO1, AMPD3, LPL

Findings which form the basis for a transcriptomic atlas to underpin future research into Endometrial Cancer.

Supplementary Table 6: List of all common DEGs in endometrial cancer in three datasets

Gene name	First Dataset (GSE115810)	Second Dataset (GSE21882)	Third dataset (GSE17025)
SFRP4	Down regulated in grade 2	Down regulated in dead	N/A
CYP1B1	Down regulated in grade 2 and grade 3	Down regulated in dead	N/A
BCLAF1	Up regulated in grade 2	Down regulated in dead	N/A
MITF	Down regulated in grade 2	Down regulated in dead	N/A
HOXA11	Down regulated in grade 2	Down regulated in dead	N/A
CORO1B	Up regulated in grade 2	Up regulated in dead	N/A
SOCS2	Down regulated in grade 2 and grade 3	Down regulated in dead	N/A
CCND2	Down regulated in grade 2	Down regulated in dead	N/A
SNCAIP	Down regulated in grade 2	Down regulated in dead	N/A
APOD	Down regulated in grade 2	Down regulated in dead	N/A
RAMP1	Down regulated in grade 2	Down regulated in dead	N/A
SPARCL1	Down regulated in grade 2	Down regulated in dead	N/A
MBNL2	Down regulated in grade 2	N/A	Down regulated in grade 3
MSH6	Down regulated in grade 3	N/A	Up regulated in grade 3
C9orf40	Down regulated in grade 2	N/A	Up regulated in grade 3
COX7A1	Down regulated in grade 2	Down regulated in dead	Down regulated in grade 3

Supplementary Table 7: List of statistically significant DEGs identified in stage I endometrial cancer from the study GSE17025

Up regulated in grade 3	MCM4, MSH6, BRIP1, ATAD2, TADA2A, UG0898H09, SOX2, WNK3
Down regulated in grade 3	DNAH6, PCAT19, LIMS4///LIMS3, ANAPC4, SCGB1D2, RNF157-AS1, VWA3A, DNAH5, MBNL2, SLC25A35, CA12, LOC101929219///LOC100505650///C1orf186, MAP3K19, LOC100288570, EPPIN-WFDC6///EPPIN, SORBS2, LOC100507334///LOC100288570///LIMS3-LOC440895///LOC440895, DNAAF1, C4orf22, ADAMTS8, ERICH3, ABHD6, LOC101929219///C1orf186, LOC388780, MAATS1, CAMK2N1, MS4A8, SCGB2A1, FOXA2, DNAH9, USP53, CAPN6

Supplementary Table 8: Number of genes differentially expressed in endometroid cells from the study GSE178671

DEGs in recurrent endometrial cancer	Up regulated: 42 DEGs<br>Down regulated: 42 DEGs
DEGs in recurrent endometrial cancer, FIGO stage IB	Up regulated: 54 DEGs<br>Down regulated: 46 DEGs

Supplementary Table 9: List of DEGs between survivor and non-survival stage 1 endometrial cancer patients in GSE21882

Genes up regulated in deceased patients	MGC2408, ECE2, SMG5, PAQR6, FOXQ1, NRAS, RAG1AP1, C1orf85, WDR51A, SLC39A1, DARS2, TNFRSF19L, KCNK5, KIF2C, NEK2, CORO1B, NA, BC111724, NUF2, ESPL1, CDKN2A, CDKN2D, RPL39L, E2F1, SLC2A1, C19orf54, HTATIP2, GINS1, CCDC77, DHX37, TCRA, TRA, hADV14S1, AV1S3A1T, SALL4, COX10, ENTPD6, CDCA3, BC043356
Genes down regulated in deceased patients	REV3L, SFRP4, TSHZ3, C14orf44, C13orf21, SF1, CGNL1, ZFP2, ZCCHC12, SMAD5, KIAA0738, ARHGEF15, CYP1B1, NA, BCLAF1, C9orf26, MITF, ISOC1, ADH5, SYTL4, LAG3, RPL15, NA, HOXA10, HOXA11, PDE6H, FLJ11200, IL6ST, PDCD4, GIMAP7, CR617391, CR592254, NR_003038, ZNF354C, PDE5A, RPL36AL, SOCS2, UBL3, HVCN1, CR933654, C3orf42, NA, PTPRV, CENPC1, CCND2, SNCAIP, BCL2, ZIM2, YTHDC1, KIAA0342, tiga1, HISPPD1, COX7A1, AHI1, ACD, RG9MTD1, DKFZp667P0924, UNQ2430, ANKRD36, KIAA1641, GSTM5, APOD, RAMP1, ATRNL1, LAPTM4A, GRIK3, FOXP2, SPARCL1, ADAMTS19,ADAMTS17, KIAA1076, AHI1, ALDH1A2

Supplementary Table 10: Comparisons used for analysis between healthy controls and cases, or different grades of tumors

Study title	Disease condition	Cell type	Analysis type	Accession	PMID
Profiles for long noncoding RNAs in ovarian granulosa cells from polycystic ovary syndrome patients with different serum concentrations of androgen [21]	PCOS<br>PCOS with hyperandrogenism<br>PCOS with normal testosterone level	Ovarian granulosa cells	Healthy controls – PCOS<br>Healthy controls – PCOS with hyperandrogenism<br>Healthy controls - PCOS with normal testosterone level	GSE106724	30224242
Cabergoline-treated lutein granulosa cells from polycystic ovarian syndrome (PCOS) patients exhibit higher transcriptomic response than cabergoline-treated controls [20]	PCOS	Lutein granulosa cells (LGCs)	Healthy controls – PCOS patients (non-treated)	GSE98595	29439093
Theca cell gene expression [22]	PCOS	Theca cells	Healthy controls – PCOS (Platform: U133A)<br>Healthy controls – PCOS (Platform: U133B)	GSE1615	15598877
Expression data from human cumulus cells isolated from oocytes at MI and MII stages in polycystic ovary syndrome (PCOS) patients [23]	PCOS	Oocytes - Cumulus cells	PCOS stage metaphase 1(MI) – PCOS stage metaphase II (MII)	GSE40400	23603633
Mesenchymal Stem/ Progenitors and Other Endometrial Cell Types from Women with Polycystic Ovary Syndrome (PCOS) Display Inflammatory and Oncogenic Potential [24]	PCOS	Endometrium - Epithelial cells	Healthy controls (Proliferative stage) – PCOS patients	GSE48301	23824412

Gene expression profiling in skeletal muscle of PCOS after pioglitazone therapy [18]	PCOS	Skeletal muscle	Healthy controls – PCOS patients	GSE8157	18560589
Reduced expression of mitochondrial oxidative metabolism genes in skeletal muscle of women with PCOS [19]	Obese PCOS (with insulin resistance)	Skeletal muscle	Healthy controls – PCOS patients	GSE6798	17563058
Expression profiling of endometrial cancer of different grades [28]	Endometrial cancer	Endometrial cancer	Control – Endometrial cancer patients<br>Control – Endometrial cancer grade 2 patients<br>Control – Endometrial cancer grade 3 patients	GSE115810	31795319
Gene Expression Analysis of Stage I Endometrial Cancers [26]	Stage I Endometrial cancer	Endometrial cancer	Grade 1 Endometrial cancer – Grade 3 Endometrial cancer	GSE17025	21619611
A 4-gene signature associated with recurrence risk in patients with low and intermediate-risk endometrial cancer [25]	Endometrial cancer	Endometrial cancer	Non-recurrent cancer – Recurrent cancer patients<br>Non-recurrent cancer FIGO staging IB – Recurrent cancer patients FIGO staging IB	GSE178671	34485158
Identification of a gene expression signature for survival prediction in type I endometrial carcinoma [27]	Stage I Endometrial cancer	Endometrial cancer	Survivors – Non-Survivors	GSE21882	20635577

Supplementary File 1: Intersections. See Supplementary File 1.

Supplementary File 2: GSE48301. See Supplementary File 2.

Supplementary File 3: GSE106724. See Supplementary File 3.

Supplementary File 4: GSE98595. See Supplementary File 4.

Supplementary File 5: GSE1615. See Supplementary File 5.

Supplementary File 6: GSE40400. See Supplementary File 6.

Supplementary File 7: GSE8157. See Supplementary File 7.

Supplementary File 8: GSE6798. See Supplementary File 8.

Supplementary File 9: GSE115810. See Supplementary File 9.

Supplementary File 10: GSE17025. See Supplementary File 10.

Supplementary File 11: GSE178671. See Supplementary File 11.

Supplementary File 12: GSE21882. See Supplementary File 12.
